# Validity and interpretation of spirometric recordings to diagnose COPD in UK primary care

**DOI:** 10.2147/COPD.S133891

**Published:** 2017-06-07

**Authors:** Kieran J Rothnie, Joht S Chandan, Harry G Goss, Hana Müllerová, Jennifer K Quint

**Affiliations:** 1 Respiratory Epidemiology, Occupational Medicine and Public Health, National Heart and Lung Institute, Imperial College London; 2 Faculty of Epidemiology and Population Health, London School of Hygiene and Tropical Medicine, London; 3 Queen Elizabeth Hospital Birmingham, University Hospitals Birmingham, Birmingham; 4 Medical School, Faculty of Medical Sciences, University College London; 5 Jersey General Hospital, St Helier, Jersey; 6 Respiratory Epidemiology, GlaxoSmithKline R&D, Uxbridge, UK

**Keywords:** pulmonary disease, chronic obstructive, general practice, respiratory function tests, data accuracy, electronic health records

## Abstract

**Background:**

The diagnosis of COPD is dependent upon clinical judgment and confirmation of the presence of airflow obstruction using spirometry. Spirometry is now routinely available; however, spirometry incorrectly performed or interpreted can lead to misdiagnosis. We aimed to determine whether spirometry undertaken in primary care for patients suspected to have COPD was of sufficient quality and whether their spirometry was correctly interpreted.

**Methods:**

Two chest physicians re-read all spirometric readings for both quality of the procedure and interpretation, received as a part of COPD validation studies using data from the Clinical Practice Research Datalink (CPRD). We then used logistic regression to investigate predictors of correct interpretation.

**Results:**

Spirometry traces were obtained for 306 patients, of which 221 (72.2%) were conducted in primary care. Of those conducted in primary care, 98.6% (n=218) of spirometry traces were of adequate quality. Of those traces that were of adequate quality and conducted in primary care, and in whom a general practitioner (GP) diagnosis of COPD had been made, 72.5% (n=218) were consistent with obstruction. Historical records for asthma diagnosis significantly decreased odds of correct interpretation.

**Conclusion:**

The quality of the spirometry procedure undertaken in primary care is high. However, this was not reflected in the quality of interpretation, suggesting an unmet training in primary care. The quality of the spirometry procedure as demonstrated by spirometric tracings provides a re-assurance for the use of spirometric values available in the electronic health care record databases for research purposes.

## Introduction

COPD and exacerbations of COPD represent an enormous health burden worldwide. Currently, COPD is the third leading cause of death worldwide.^1^ In England and Wales alone, ~25,000 people a year die of COPD, and between 2007 and 2009, COPD accounted for 4.8% of all deaths in England.^2^ There are ~1.2 million people living with COPD in the UK.^3^

There is no single diagnostic test for COPD. The diagnosis relies on clinical judgment based on a combination of history, physical examination, and confirmation of the presence of airflow obstruction using spirometry.^4^ Spirometry is now more routinely available and is used as standard to determine severity of airflow limitation in COPD patients in epidemiological studies rather than drug treatments as a proxy, which have previously been used.^5^ However, spirometry if incorrectly performed or interpreted can lead to misdiagnosis (both over and under diagnosis of COPD). Common errors in the performance of spirometry that would impact on quality and therefore interpretability include, for example, submaximal inhalation, variable effort, extra breaths, or coughing during the procedure. Common possible errors in interpretation include not appreciating that the quality is insufficient or can arise from the use of inappropriate reference values. Previous work conducted over a decade ago, before the introduction of the Quality and Outcomes Framework (QOF), found that both the performance and the interpretation of spirometry for the diagnosis of COPD in UK primary care were low.^6^ Over half of those with a diagnosis of COPD had significant reversibility of obstruction (range 210–800 mL), and almost one-third had normal lung function.^6^ Another study conducted in the UK primary care in 2007 further concluded that the quality of conduct of spirometry in primary care was also low (32% of tests were of unacceptable quality).^7^

Knowing that primary care electronic health care record (EHR) spirometry data are accurate is important as many respiratory diseases are diagnosed and managed in primary care. Additionally, observational research can use data entered into EHR both as single measurements to determine severity of airflow obstruction for study population description and statistical modeling as a covariate and to track changes in lung function over time as disease progression outcomes. In a recent COPD validation study in Clinical Practice Research Datalink (CPRD), it was found that use of confirmation of COPD diagnosis by spirometry did not greatly improve the validity of the definition of COPD in the CPRD, thus raising questions about the validity and interpretation of spirometry in this setting.^8^

Using data from the CPRD, we aimed to determine whether spirometry undertaken in primary care for patients suspected to have COPD was of sufficient quality and whether the spirometry was subsequently correctly interpreted.

## Methods

### Data sources

The CPRD is a large, longitudinal electronic database of primary care medical records that contains anonymized records for >13 million patients, of whom 4.4 million are currently registered with a practice that is contributing data to the CPRD, representing ~9% of the UK population.^9^ Data held include information on consultations, diagnoses, tests (including spirometry), and referrals to secondary care and prescriptions from primary care and some lifestyle data. Data are predominately recorded using a system of “Read codes”, a hierarchical system of codes that describe multiple phenomena, including diagnoses, clinical signs, symptoms, and lifestyle characteristics. Additionally, actual test values are recorded in some fields under the heading of entity types.

### Study population

As part of previous studies to validate the recording of COPD diagnosis and exacerbations of COPD in CPRD,^8^,^10^ along with a questionnaire, general practitioners (GPs) were asked to send additional information (including spirometry traces) that may have supported or refuted a diagnosis of COPD. GPs also indicated whether they thought the patient had COPD and if the patient had received a diagnosis of any other respiratory disease. Patients were only included in this study if the additional supporting information sent by GPs included spirometry traces. We were able to obtain further clinical and demographic characteristics of these patients from their CPRD record. Data were “twice encrypted” within CPRD to ensure anonymity, first between practices and CPRD and second from CPRD to researchers.

### Assessment of quality and interpretation of spirometry

For both the previous validation studies, two respiratory physicians (JKQ and JRH) assessed all available spirometry traces for 1) quality and 2) diagnostic interpretation. Spirometry quality was judged by examining the flow volume loop according to American Thoracic Society/European Respiratory Society (ATS/ERS) criteria.^11^ Briefly, spirometry was judged to be of low quality if it had any of the following features: insufficient expiratory time (<6 seconds), incomplete expiration (determined by absence of volume–time plateau from the flow volume loop), evidence of coughing, expiration was too slow (determined from the flow volume loop), or there was no evidence of reproducibility. We followed ATS/ERS guidelines for repeatability criteria: three technically acceptable results should be selected from up to five efforts, repeatability criteria that are met when there is no >100 mL ideally (and certainly no >150 mL in the occasional highly variable patient) between each blow.

Spirometry results were classified by an independent respiratory physician (JKQ) as 1) normal, 2) indicative of obstruction, 3) indicative of restriction, or 4) mixed obstruction and restriction. We used the ATS/ERS guidelines to guide interpretation.^12^ Obstruction was defined as expiratory volume in one second (FEV_1_)/forced vital capacity (FVC) ratio < lower limit of normal (LLN). Restriction was defined as FVC <85% predicted and FEV_1_/FVC ≥0.55. Those with both obstruction and restriction were categorized as having a mixed defect. Those traces with no evidence of obstruction or restriction were considered to be normal. We did not assess reversibility. Interpretation of spirometry by health practitioners in the primary care setting was obtained from questionnaires, and additional material returned with questionnaires. Diagnostic interpretation of the spirometry traces by the respiratory physician was taken to be the reference standard, and interpretations of health practitioners in the primary care setting were compared to this. We were unable to tell who within the GP practice had actually performed the spirometry.

### Analysis

The primary analysis focused on the accuracy of identification of a COPD diagnosis in the presence of a valid spirometry trace. Logistic regression was used to assess predictors of primary care health practitioner interpretation of spirometric traces with the outcome of COPD diagnosis confirmed or not confirmed by respiratory physician adjudication of spirometry traces. Age, sex, and previous record for asthma diagnosis were covariates. Additionally, bronchodilators before spirometry status was used as a covariate in a sensitivity regression model restricted to those where it was clear from the spirometry report if it was conducted pre- or post-bronchodilator (n=78).

### Ethics

Ethical approval was obtained from the London School of Hygiene and Tropical Medicine (LSHTM) Observational Research Ethics Committee (approval numbers 6481 and 6204) and the CPRD Independent Scientific Advisory Committee (ISAC) (approval number 12_065A). Patient records and questionnaire responses were de-identified and anonymized by CPRD staff before being sent to the investigators. The ISAC protocol is available on request.

## Results

**Figure 1:**
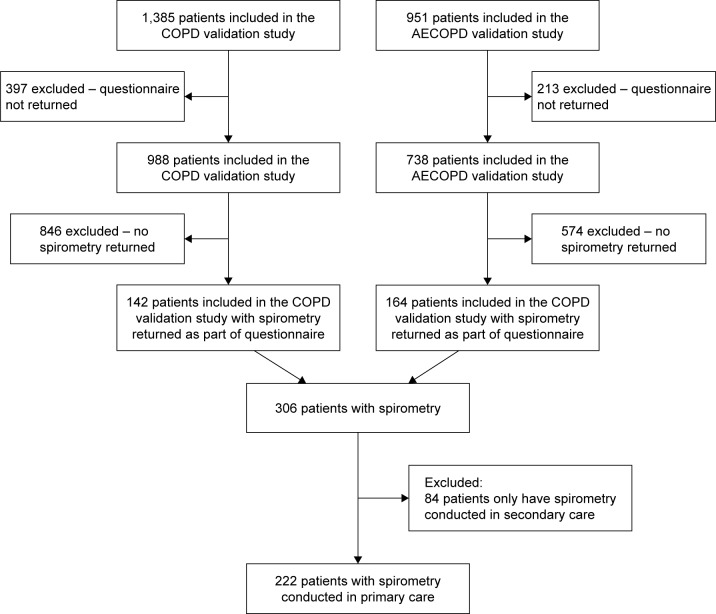
Flow diagram of patient entry into the study (n=222). **Abbreviation:** AECOPD, acute exacerbation of COPD.

Spirometry traces were obtained for 306 patients, of which 230 (75.1%) were conducted in primary care (Figure 1). The characteristics of the patients included in the study are presented in Table 1. Briefly, 47.7% were female; the mean age was 63.1 years (standard deviation [SD] 10.0). The sample was evenly split among males and females and among ex-smokers and current smokers. Almost 30% had a previous GP diagnosis of asthma.

In total, 56.7% (n=161) of the traces were obtained as part of the acute exacerbation of COPD (AECOPD) validation study and the remaining as part of the COPD validation study.

Of those conducted in primary care, 96.5% (n=222) of spirometry traces were of adequate quality such that a valid interpretation could be made.

Of those traces that were conducted in primary care and were of acceptable quality, 27.9% (n=62) of the traces were definitely conducted post-bronchodilator and 7.2% (n=16) were definitely conducted pre-bronchodilator. For the remaining (64.9%, n=144), it was unclear if spirometry was conducted pre- or post-bronchodilator.

Of those traces that were of adequate quality and conducted in primary care, and in whom a GP diagnosis of COPD had been made, 72.5% (158) of the spirometry traces labeled as COPD were consistent with obstruction (Table 2).

Regression models indicated that correct interpretation of spirometry (as obstructive, restrictive, or normal) was influenced by a record for a previous asthma diagnosis (odds ratio [OR] 0.49, 95% confidence interval [95% CI] 0.26–0.93). There was no evidence that correct interpretation was influenced by age, sex, or whether the spirometry was conducted pre- or post-bronchodilator (Table 3).

## Discussion

We found the quality of spirometry undertaken in primary care to be high (>96% had acceptable quality), with gaps in validity of interpretation in primary care. This suggests a large improvement in the quality of spirometry; however, it seems that the validity of interpretation of spirometry has improved only modestly over the last decade.

### Strengths and limitations

One of the strengths of this work is the representativeness of the CPRD database. Our findings are also strengthened by the fact we were able to review actual traces. However, we could not always tell whether traces were performed pre- or post-bronchodilator, again highlighting a clinically important area in terms of coding in records. This limitation means that we could not stratify patients into pre- and post-bronchodilator for the analysis. In the UK, patients with COPD should have spirometry conducted every 15 months. As such, it is likely that most traces were conducted in people who the GP has already diagnosed with COPD. A substantial proportion of these patients will be using long-term bronchodilators, and as such, their spirometry will be “post-bronchodilator”. We appreciate also that our responders might not be truly representative of all GPs and we could not tell who within the practice was actually performing and interpreting the spirometry traces.

### Comparison with existing literature

Compared to work conducted over a decade ago,^6^ we found a large improvement in the quality of spirometry conducted in primary care; however, the proportion of those with correctly interpreted spirometry was not markedly improved. Previous work has suggested that age and sex influenced interpretation of spirometry for diagnosis of COPD.^13^ We did not find that age or sex influenced accuracy of interpretation; this may have been due to lower power in our study to detect these differences. We found that a previous asthma diagnosis, however, decreased the probability that a valid interpretation would be made.

### Implications for research and practice

Our results suggest that when undertaking research in UK primary care databases, such as CPRD, it is better to use actual values recorded for spirometry rather than relying on the interpretation Read codes entered into the patient’s record. In our previous study that validated the recording of COPD in UK EHR, we found that by using a diagnostic code for COPD combined with a smoking history resulted in an algorithm for the ascertainment of COPD that had a high positive predictive value.^8^ Although we did not investigate the utility of addition of an obstructive spirometric ratio to the algorithm, when we assessed the addition of a marker indicating whether or not spirometry was performed, this did not improve the positive predictive value. This suggested that interpretation of spirometry for the diagnosis of COPD might be less than ideal, a finding reflected in the results from this study. Our results are also important clinically, as they indicate an unmet training need within primary care in the interpretation of spirometry results.

According to guidelines for clinical care, COPD patients should have their diagnosis confirmed by spirometry within 12 months of diagnosis and should have their FEV_1_ monitored yearly. Both of these recommendations are incentivized by QOF. This means that the CPRD is potentially a rich source of valid longitudinal information on spirometry results. FEV_1_ is an important outcome in COPD studies. Demonstrating the validity of FEV_1_ values in CPRD means that researchers can use this resource to study FEV_1_ as an outcome in COPD studies, such as those investigating COPD disease epidemiology or the effects of interventions.

## Conclusion

Spirometry is performed in primary care to a high standard. However, interpretation of spirometry in patients with suspected COPD in primary care is moderate. Entered values from spirometry are valid and can be used for research. Efforts should be made to improve spirometry interpretation for high-quality patient care.

## Figures and Tables

**Table 1:** Characteristics of included patients with interpretable spirometry conducted in primary care (n=222)

Characteristics	n (%)
Age group, years	
<55	52 (23.4)
55–64	81 (36.5)
65–74	63 (28.4)
75+	26 (11.7)
Sex	
Male	117 (52.7)
Female	105 (47.3)
Smoking status	
Ex-smoker	106 (47.8)
Current smoker	116 (52.3)
Previous GP diagnosis of asthma	
Yes	67 (30.2)
No	155 (69.8)

**Abbreviation:** GP, general practitioner.

**Table 2:** Respiratory physician interpretation of spirometry for patients diagnosed with COPD in primary care (n=218)

Respiratory physician spirometry interpretation	n (%)
Normal	52 (23.4)
Obstructive	159 (71.6)
Restrictive	9 (4.1)
Mixed obstructive and restrictive	2 (0.9)

**Table 3:** Predictors of correct interpretation of valid spirometry traces carried out in primary care (n=222)

Characteristics	Crude OR (95% CI)	Adjusted OR (95% CI)*
Age (per year)	0.98 (0.96–1.01)	0.98 (0.96–1.01)
Female sex	1.26 (0.69–2.30)	1.28 (0.69–2.38)
Previous GP diagnosis of asthma	0.50 (0.27–0.94)	0.49 (0.26–0.93)
Spirometry conducted post-bronchodilator**	0.62 (0.20–2.03)	0.61 (0.18–2.09)

**Notes:**

* Adjusted for other characteristics in the table.

** Reference category is spirometry conducted pre-bronchodilator. Excluding traces where it was unclear if spirometry was conducted pre- or post-bronchodilator.

**Abbreviations:** OR, odds ratio; CI, confidence interval; GP, general practitioner.
